# Anti-aging effects exerted by Tetramethylpyrazine enhances self-renewal and neuronal differentiation of rat bMSCs by suppressing NF-kB signaling

**DOI:** 10.1042/BSR20190761

**Published:** 2019-06-25

**Authors:** Xiaoqing Song, Jin Dai, Huaguang Li, Yuemeng Li, Weixiao Hao, Yu Zhang, Yuping Zhang, Lining Su, Huiping Wei

**Affiliations:** 1 Biology Office, Basic Medical College of Hebei North University, Zhangjiakou, Hebei 075000, P.R. China; 2 Department of Ophthalmology, Mengcun Hui Autonomous County Hospital, Cangzhou, Hebei 061400, P.R. China; 3 Department of Orthopaedics, First Affiliated Hospital of Hebei North University, Zhangjiakou, Hebei 075000, P.R. China

**Keywords:** Mesenchymal stem cells, neuronal differentiation, NF-κB signaling, proliferation, senescence, Tetramethylpyrazine

## Abstract

In order to improve the therapeutic effects of mesenchymal stem cell (MSC)-based therapies for a number of intractable neurological disorders, a more favorable strategy to regulate the outcome of bone marrow MSCs (bMSCs) was examined in the present study. In view of the wide range of neurotrophic and neuroprotective effects, Tetramethylpyrazine (TMP), a biologically active alkaloid isolated from the herbal medicine *Ligusticum wallichii*, was used. It was revealed that treatment with 30–50 mg/l TMP for 4 days significantly increased cell viability, alleviated senescence by suppressing NF-κB signaling, and promoted bMSC proliferation by regulating the cell cycle. In addition, 40–50 mg/l TMP treatment may facilitate the neuronal differentiation of bMSCs, verified in the present study by presentation of neuronal morphology and expression of neuronal markers: microtubule-associated protein 2 (MAP-2) and neuron-specific enolase (NSE). The quantitative real-time polymerase chain reaction (qRT-PCR) revealed that TMP treatment may promote the expression of neurogenin 1 (Ngn1), neuronal differentiation 1 (NeuroD) and mammalian achaete–scute homolog 1 (Mash1). In conclusion, 4 days of 40–50 mg/l TMP treatment may significantly delay bMSC senescence by suppressing NF-κB signaling, and enhancing the self-renewal ability of bMSCs, and their potential for neuronal differentiation.

## Introduction

Mesenchymal stem cells (MSCs) are a kind of adult stem cells derived from the mesodermal germ layer, and it exists in bone marrow [1], adipose tissue [2], amniotic fluid [3], umbilical cord blood [4], placenta [5], and even menstrual blood [6]. Under the appropriate conditions, MSCs can differentiate into a wide range of cell types, such as neural cells [7], myocytes [8], hepatocytes [9], endothelial cells [10], and others. In addition, MSCs exhibit low expression of major histocompatibility complex (MHC)-I molecules and are negative for MHC-I [11], which is beneficial for the success of autologous and allogeneic transplantation. In view of their aforementioned advantages, MSCs are considered an ideal candidate for repairing or regenerating desired tissue at present [12,13]. However, a series of researches revealed the self-renewal ability and multipotency of MSCs would decline and even lose along the consecutive passage *in vitro*, during which cellular senescence has been suggested to occur [14–18]. And cellular senescence further impedes the therapeutic potential of MSCs following MSC transplantation in diseased regions.

As one of the main bioactive components extracted from the Chinese herb Chuanxiong, tetramethylpyrazine (TMP), a para-dihydroxy derivative of ligustrazine [19,20], has been reported to exhibit a neuroprotective effect [21–23], in addition to an anti-inflammatory [18,24] and anti-aging effect [18,21,25] *in vivo*, and therefore, is widely used to reduce ischemic brain injury [26,27] and acute spinal cord injury [28]. Nowadays, a large number of researches have demonstrated that TMP serves promoting roles in proliferation [29], in differentiation of cells cultured *in vitro* [25,30], and in protection of cells from oxidative damage [31]. However, the findings of a number of studies are not in concordance with the aforementioned. For instance, Bi et al. [32] revealed that TMP-induced cell cycle arrest at the G_0_/G_1_ checkpoint and promoted a caspase activation-dependent mitochondrial apoptosis. Shen et al. [33] reported TMP significantly inhibited cell viability, migration, and invasion rate, and increased apoptosis by reducing Akt activity and by increasing the activity of caspase-3.

Based on these previous researches, it was estimated that the positive or negative effects of TMP on cell proliferation, lineage commitment, and apoptosis largely depended on its dosage and the duration of incubation with cells. To investigate this hypothesis, TMP was employed at different concentrations to culture rat bone marrow MSCs (bMSCs), and at various time points, cell viability, senescence, proliferation, cell cycle, and neuronal differentiation were examined to screen the appropriate TMP dosage and culture time. Meanwhile, the activity of NF-κB signaling in bMSCs exposed to TMP was monitored at different concentrations. Previous studies have revealed that the activation of NF-κB signaling accelerated tissue and cellular senescence [34,35].

NF-κB, an important transcription factor (TF), is composed of five distinct subunits in mammalian cells: RelA (p65), RelB (p50), c-Rel, NFKB1, and NFKB2. It is usually inactive and is sequestered by endogenous inhibitor protein IκB [36]. The activation or inactivation of NF-κB depends to a great extent on IκB kinase (IKK). IKK consists of two catalytic subunits, IKKα/IKK1 and IKKβ/IKK2, and one regulatory subunit IKKγ/NEMO, serving a crucial role in regulating cell aging [37]. A series of pro-inflammatory factors, such as tumor necrosis factor (TNF) α (TNF-α) and interleukin (IL)-1β, have been indicated to activate the NF-κB signal pathway [38]. Once stimulated by these factors, adaptor proteins, including TRADD and TRAFs, acting as E3 ubiquitin ligases, have been reported to lead to ubiquitinoylation and degradation of NEMO [39], and meanwhile, TGF-β-activated kinase-1 (TAK1) [40] can be activated upon the stimulation of the pro-inflammatory factors, which further phosphorylates the catalytic subunit IKKβ. The phosphorylated IKKβ (p-IKKβ) in turn catalyzes the phosphorylation of IκB and p65 subunit. Rap1 acts as a regulator of the NF-κB signaling by promoting IKK-mediated phosphorylation of p65, leading to activation of NF-κB target genes, which was confirmed by overexpression and/or knockout of *Rap1* gene [41–42] has verified that Rap1 plays a vital role in regulating NF-κB signaling, during which Rap1 can stabilize IKK by its direct binding to IKKα and p-IKKβ, and trigger p65 release [42–43]. Hereby, p65 and p50 are subsequently transferred into the nucleus to promote the expression of its downstream target genes (such as inducible nitric oxide synthase, cyclooxygenase-2, and others), and further trigger cellular senescence [38,39]. Therefore, the p-IKKβ and the phosphorylated p65 (p-p65) are regarded as activation markers of the NF-κB signaling pathway [44].

In conclusion, the aim of the present study is to promote TMP application in the field of neuronal regeneration by modifying MSCs, and to improve the therapeutic effects of MSC-mediated therapy.

## Materials and methods

### Animals and bMSC culture

Three Sprague–Dawley (SD) rats 3–4 weeks old were purchased in Animal Center of Hebei North University. All animal treatments were performed under the unconsciousness by euthanasia with sodium amytal (intraperitoneal injection, 50 mg/kg body weight) to minimize animal suffering. The rat hind legs were dismembered, and bMSCs were isolated from the femurs. After experiment the dead bodies of animals were treated by the Experimental Animal Center of Hebei Northern University. All animal experiments were approved by the Animal Ethics Committee of Hebei North University (Approval Number: 2018-1-9-05). Cell culture was conducted using low Dulbecco’s modified Eagle’s medium (L-DMEM) (Gibco) and 10% fetal bovine serum (FBS; Gibco). All cells were seeded into culture flasks and cultured at 37°C with 5% CO_2_. Once the cell monolayer reached 80–90% confluence, the cells were trypsinized with 5% trypsin for subculture.

In order to exhibit the morphology of bMSCs clearly, Diff-Quick staining agent (Beijing Propbs Biotechnology Co., Ltd.) was used to stain the bMSCs at passage 4 (P_4_), in accordance with previous research [45]. Following washing with PBS, images were obtained under the multifunctional fluorescent microscope (×200) (Eclipse 90i; Tokyo Nikon Corporation).

### Cell authentication

Cell authentication was performed through immunofluorescence staining and flow cytometry (FCM) (Becton-Dickinson, U.S.A.) equipped with FACSDiva analytical 8.0.1. software (BD Biosciences). Briefly, bMSCs (10^6^ cells/ml, 1 ml) at P_4_ were respectively incubated with the following antibodies (1 μl): Fluorescein isothiocyanate (FITC)-IgG, allophycocyanin (APC)-CD90 and FITC-CD45 (BD Bioscience) for 30 min in the dark at room temperature. Positive cells were detected by FCM. Meanwhile, cells were seeded onto the culture slides and immunofluorescence staining was performed. When cells reached 80–90% confluence, the cells were fixed with 4% paraformaldehyde for 15 min and blocked with 2% bovine serum albumin (BSA; Shanghai Sangon Biotech Co., Ltd.) for 1 h at room temperature. And subsequently, cells were stained with the aforementioned antibodies in the dark for 30 min at room temperature. Following PBS wash, images were captured under a fluorescent microscope. The cells that were stained with FITC-IgG were regarded as the isotype control.

### TMP treatment and cell viability assay

Following cell authentication, the subculture was carried out until bMSCs got up to passage 7 (P_7_). bMSCs at P_7_ were subsequently cultured using conditioned media consisting of complete medium and different TMP concentrations (0, 20, 30, 40, 50, 60, 70 and 80 mg/l) for 1, 2, 4, 6, and 8 days. The medium containing 0 mg/l TMP (purity > 98%; Shanghai Meryer Chemical Technology Co. Ltd.) was the complete medium and was used as the control.

In order to screen a suitable TMP concentration and incubation time, the cell counting kit-8 (CCK-8) kit (Shanghai Yeasen BioTech Co., Ltd.) was utilized to detect cell viability, according to the manufacturer’s protocols. Cells (1 × 10^3^) in 100 μl complete medium were plated in 96-well plates per well. Subsequent to the cells adhering to the culture plates, the used medium was replaced with the conditioned media, and incubation was maintained for the duration of the aforementioned time points. Following cells being rinsed with PBS in duplicate, 10 μl CCK-8 solution in 100 μl L-DMEM was added and incubation was maintained for an additional 2 h at 37°C with 5% CO_2_. The optical density (OD) values were measured with a microplate reader (Bio-Rad Laboratories, Inc., U.S.A.) at a wavelength of 450 nm.

### Senescence-associated β-galactosidase staining

The increased senescence-associated β-galactosidase activity (SA-β-gal) is a consequence of the senescence [46], and SA-β-gal staining was regarded as one of the best-characterized and most convenient methods to measure cell senescence. SA-β-gal staining kit (Shanghai Beyotime Institute of Biotechnology, Co., Ltd.) was used to determine the aging situation of bMSCs, according to the manufacturer’s protocols. A total of 200 μl bMSCs at P_7_ were seeded in 24-well plates at a density of 5 × 10^3^ cells/well and cultured for 4 days in their respective media. At room temperature, cells were fixed with 4% polyformaldehyde for 15 min at ∼60% confluence. Subsequently, SA-β-gal staining solution (10 μl solution A, 10 μl solution B, 930 μl solution C, and 50 μl X-Gal reagent) was added and incubated overnight at 37°C in a water bath. Blue color represented the SA-β-gal-positive cells. The senescent rate was counted under a phase-contrast microscope (Eclipse 90i; Nikon).

### NF-κB signaling analysis using Western blot assay

Western blot assay was used to analyze the protein quantities of p-IKKβ and p-p65. A total of 200 μl bMSCs were sown in six-well plates at a density of 5 × 10^4^ cells/ml. Following adherence culture, bMSCs were treated with TMP as aforementioned, from which total protein was extracted using RIPA lysis buffer (Beijing Applygen Technologies, Inc.). The bicinchoninic acid assay kit (Shanghai BestBio Biotech, Co., Ltd.) was used, according to the manufacturer’s protocols to determine the protein concentration. Protein (15 μg) from each sample mixed with loading buffer was loaded on to the 12% odium dodecyl sulfate/polyacrylamide gel electrophoresis (SDS/PAGE). Following electrophoresis, protein bands were electrotransferred on to the polyvinylidene difluoride (PVDF) membranes and the membranes were blocked with 10% dry milk for 1 h at room temperature. In addition, incubation with the rabbit anti-rat primary antibodies (dilution, 1:2000; Beijing Bioss Biotechnology Inc.): p-IKKβ, p-p65 and anti-β-actin was maintained overnight at 4°C in a refrigerator. Subsequently, the membranes were washed in triplicate with PBS-T (PBS with 0.1% Tween-20), and incubated subsequently with HRP–conjugated goat anti-rabbit secondary antibody (dilution, 1:3000; Bioss) for 1 h at room temperature. Finally, the PVDF membranes were visualized using enhanced chemiluminescence detection reagent (Beyotime), according to the manufacturer’s protocols, for 3 min at room temperature in the dark. The visualization of protein bands was achieved through Gel Imaging System (Aplegen; Gel Co., Inc., U.S.A.). The band densities were selected between three and five independent blots representing the content of each protein sample. The amount of β-actin was used as the internal control.

### Detection of pro-inflammatory factors by enzyme-linked immunosorbent assay

Enzyme-linked immunosorbent assay (ELISA) kits (Wuhan Mskbio., Co., Ltd.) were used to detect the quantities of TNF-α and IL-1β secreted into the cell supernatant. The bMSCs were exposed to the same volume of condition media for 4 days. A total of 100 μl cell supernatant per group was added into the coated wells and incubated at 37°C for 2 h. Subsequently, the wash buffer was used to rinse the reaction plate five times. Thereafter, the first antibody working solution (100 μl) was added into the reaction wells, maintained for 1 h at 37°C, and washed as aforementioned. A total of 100 μl enzyme–conjugated antibody working solution was added, kept at 37°C for 30 min, and subsequently washed. In addition, 100 μl substrate working solution was added at 37°C for 15 min. Finally, 100-μl stop solution was added. Following 30 min of incubation, the OD values were determined using a microplate reader at a wavelength of 450 nm.

### EdU incorporation assay

EdU Imaging kit (Nanjing KeyGen Biotech Co., Ltd.) was used, according to the manufacturer’s protocols to detect TMP roles in bMSC proliferation. A total of 200 μl bMSCs at P_7_ were seeded in 24-well plates (7 × 10^3^ cells/well). After 6 h of adherence culture, bMSCs were exposed to TMP treatment as stated above. A total of 10 μl 5-ethynyl-2′-deoxyuridine (EdU) (20 μM) was added and incubated for 24 h at 37°C with 5% CO_2_. The cells were fixed with 4% polyformaldehyde for 15 min at room temperature. kFluor488 azide reagent and Hoechst33342 (0.5 μg/ml; Beyotime) were used to stain the nuclei for 15 min at room temperature. The images were captured under a fluorescence microscope.

### Cell cycle detection

The bMSCs at P_7_ were incubated with conditioned media for 4 days prior to propidium iodide (PI; Beyotime) staining. The cells were subsequently collected and rinsed two times with PBS. Following centrifugation at 1500 rpm for 5 min at room temperature, the supernatant was discarded. And then the cells were treated to fixation with 2 ml pre-cooled 70% ethanol, 1500 rpm centrifugation for 5 min and PBS wash for three times. Thereafter, 20 μg/ml RNase A solution was added and incubated at 37°C for 30 min. Following PBS wash and centrifugation, cell pellet was re-suspended using 50 μg/ml PI working solution and incubated for 30 min in the dark at room temperature. At last, cell cycles were analyzed through FCM.

### Neuronal differentiation and immunocytochemistry staining

After 4 days of TMP pretreatment, bMSCs were induced to differentiate into neuron-like cells using neuronal induction medium (NIM), composed of DMEM/F12 (Gibco), plus 20 μg/l nerve growth factor (NGF; Wuhan Hiteck Biological Pharma, Co., Ltd.) and 20 μg/l brain-derived neurotrophic factor (BDNF; Sangon). The differentiation differences among these TMP concentrations were compared on days 1, 3, 5, and 7 following induction.

For identification of the differentiated neuron-like cells, immunocytochemistry staining was performed. At room temperature, the differentiated cells were fixed with 4% polyformaldehyde for 15 min, and permeabilized with 0.3% (v/v) Triton-X 100 (Beyotime) for 30 min. Subsequently, 3% H_2_O_2_ was used to inactivate endogenous peroxidase for 10 min and 5% BSA was used to block non-specific proteins for 1 h. The primary rabbit anti-rat antibodies (dilution, 1:500; Bioss), Neuron-specific enolase (NSE) and Microtubule-associated protein-2 (MAP-2) were added and incubated overnight at 4°C. In addition, the cells were rinsed three times with PBS and incubated with Biotin–conjugated goat anti-rabbit secondary antibodies (dilution, 1:500; Bioss) for 1 h at room temperature. Subsequent to PBS rinse in triplicate, strept avidin–biotin complex (SABC; Wuhan Boster Biological Technology, Co., Ltd.) reagent was added and the mixture was incubated for 20 min at room temperature. At last, the DAB method was used for staining the positive cells.

### To detect the expression profiles of neuronal basic helix-loop-helix factors

To further estimate the effects of TMP on neuronal differentiation of bMSCs, the expression changes of neuronal basic helix–loop–helix (bHLH) factors, including neurogenin 1 (Ngn1), mammalian achaete–scute homolog 1 (Mash1) and neuronal differentiation 1 (NeuroD) were measured using quantitative real-time polymerase chain reaction (qRT-PCR). Following exposure to NIM for 1, 3, 5, and 7 days, the cell samples were collected, from which total RNA was extracted using TRNzol reagent (Beijing Tiangen Biotech Co., Ltd.), according to the manufacturer’s protocol. The extracted RNA was suitable for use when the OD values were between 1.9 and 2.0 at a wavelength of 260/280 nm. A total of 2 μg total RNA was reverse transcribed into cDNA using the First-Strand cDNA Synthesis kit (Tiangen), according to the manufacturer’s protocol. The synthesized cDNA served as the template for qRT-PCR. The qRT-PCR amplifications were performed using the Premix Taq PCR kit (Dalian Takara Biotechnology Co., Ltd.), and their thermocycling conditions were set as follows: initial denaturation step at 94°C for 5 min, followed by 30 cycles at 94°C for 40 s, 55°C for 40 s and 72°C for 2 min, and one cycle at 72°C for 10 min. The expression levels of these three genes were normalized to β-actin. The results were expressed as fold-change compared with the control using the 2^-ΔΔ*C*q^ method [47]. The primer sequences are presented in Table 1.

**Table 1:** qRT-PCR primers and their information

Genes	Primers (5′→3′)	*T*_m_	Fragment length
*Ngn1*			
Sense	CGGCCAGCGATACAGAGTCC	59°C	191 bp
Antisense	GTACGGGATGAAGCAGGGTG		
*Mash1*			
Sense	GGCTCAACTTCAGTGGCTTC	55°C	291 bp
Antisense	TGGAGTAGTTGGGGGAGATG		
*NeuroD1*			
Sense	TCAGTTCTCAGGACGAGGA	59°C	366 bp
Antisense	AGTTCTTGGCCAAGCGCAG		
*β-actin*			
Sense	TCACCCACACTGTGCCCATCTATGA	56°C	246 bp
Antisense	CATCGGAACCGCTCATTGCCGATAG		

To verify the qRT-PCR results mentioned above, Western blot was performed, and the cell samples treated with 40 mg/l TMP were chosen. Namely after 4 days of treatment with 40 mg/l TMP, cells were exposed to NIM for 1, 3, 5 and 7 days from which protein samples were extracted for Western blot. All procedures of Western blot were the same as above, except the primary antibodies. Here, the rabbit anti-rat primary antibodies: anti-Ngn1, anti-NeuroD and anti-Mash1 (dilution, 1:500; Bioss) were used.

### Statistical analysis

SPSS 17.0 software (SPSS, U.S.A.) was used to analyze data. All data were expressed as mean ± standard deviation. Homoscedasticity of primary data was first detected using Levene’s test, and variance was demonstrated to be homogeneous. One-way analysis of variance (ANOVA) was applied to determine the significant difference among multiple groups. Pairwise comparisons between different groups were performed using Tukey’s post hoc significance test. *P*<0.05 was considered to indicate a statistically significant difference. All data were obtained from experiments performed at least in triplicate.

## Results

### bMSC morphology and Diff-Quick staining

**Figure 1:**
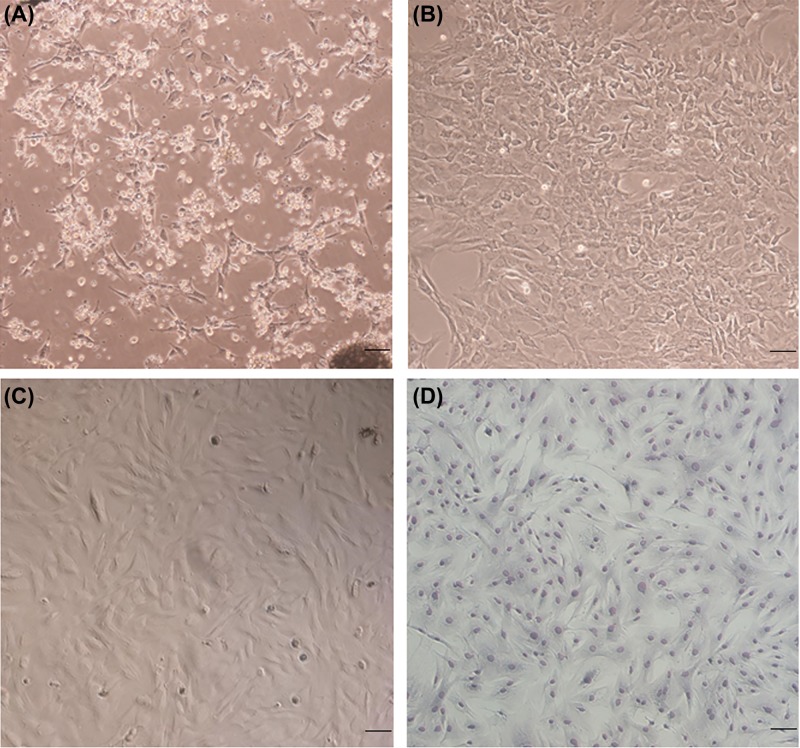
Morphological characteristics of bMSCs (**A**) Some of cells became attached after 24 h of primary culture. (**B**) The cell arrangement of primary culture. (**C**) Fourth-generation bMSCs exhibited a spindle-like morphology. (**D**) Fourth-generation bMSCs were stained with Diff-Quick solution. Scale bar, 50 μm.

Following primary culture for 24 h, a number of cells became adherent, and exhibited a triangular, spindle-shaped or fibroblast-like morphology (Figure 1A). However, once the cell monolayer reached 80–90% confluence, the cell arrangement was not orderly and cell morphology was indicated to be irregular (Figure 1B). When subcultured to P_4_, the majority of attached cells exhibited a typical spindle-like shape at >90% confluence (Figure 1C). Diff-Quick staining made the spindle-like shape more visible, and all cells exhibited a homogeneous morphology (Figure 1D).

### Cell authentication

**Figure 2:**
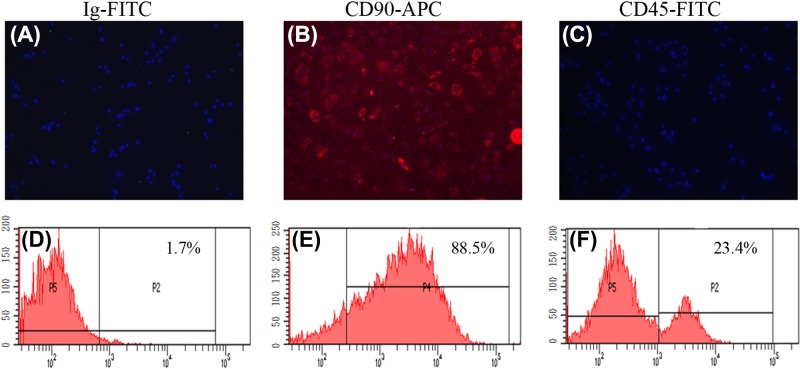
Cell authentication through immunofluorescence staining and FCM The upper panel (**A**–**C**) showed cells were respectively stained with Ig-FITC (isotype control), CD90-APC, and CD45-FITC. The lower panel (**D**–**F**) showed immunophenotypes of bMSCs analyzed by FCM; >87.82% cells were positive for CD90; and >80% were negative for CD45. Scale bar, 50 μm.

Determination of cell-surface antigen profiles of bMSCs was carried out through immunofluorescence staining and FCM. Cells stained with the isotype control antibody, FITC-IgG, were used as the control (Figure 2A,B). The results indicated that the majority of cells were positive for CD90 (Figure 2C,D) and negative for CD45 (Figure 2E,F). According to the results derived from FCM, the rate of positive cells expressing CD90 and CD45 were 87.82 ± 5.23 and 19.80 ± 4.14%, respectively.

### Cell viability assay

**Figure 3:**
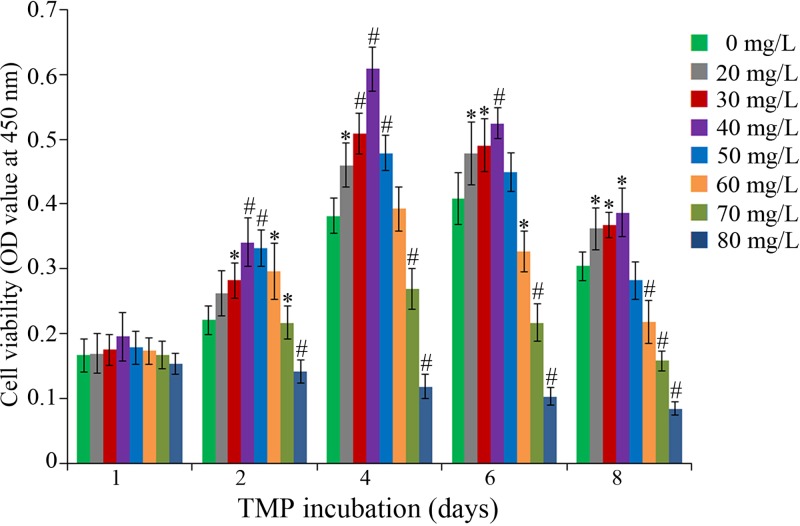
TMP exerts dose-/duration-dependent effect on the bMSC viability TMP with the concentrations of 20–60 mg/l could improve the cell viability with varying degrees after 4 days of incubation. *P<0.05 vs. control, ^#^*P*<0.01 vs. control.

CCK-8 assay revealed that compared with the control, TMP exhibited a dose- and duration- dependent effect on bMSC viability (Figure 3). In the 20–40 mg/l TMP groups, cell viability was indicated to be increasing at all aforementioned timepoints (*P<0.05 and ^#^*P*<0.01 vs. control). In particular, 4 days of incubation with 40 mg/l TMP significantly increased the viability, approximately 1.5 times higher than that in the control group. During the first 4 days, treatments with 50–60 mg/l TMP were indicated to enhance the bMSC viability; however, as the treatment progressed, the viability in these two groups began to drop (*P<0.05 and ^#^*P*<0.01 vs. control). The viability was inhibited in the 70 and 80 mg/l TMP groups from the first day (*P<0.05 and ^#^*P*<0.01 vs. control). Therefore, the experimental protocol that bMSCs at P_7_ were treated with 20, 30, 40, 50, and 60 mg/l TMP for 4 days was adopted in the following experiments.

### Assay of bMSC senescence and NF-κB signaling activity

**Figure 4:**
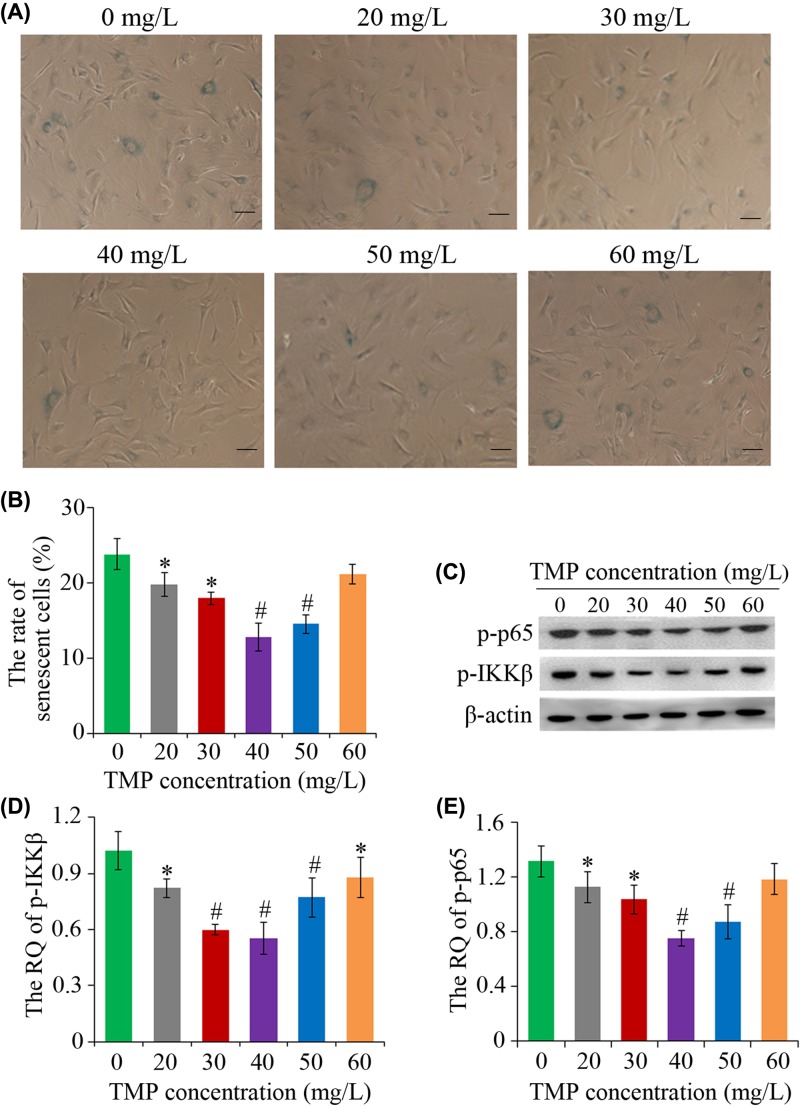
Measurement of cellular senescence using SA-β-gal staining and analysis of NF-κB signaling through Western blot (**A**) The senescent cells exhibited blue color under the phase-contact microscope. (**B**) Quantitative analysis of the senescent bMSCs. (**C**) Western blot results of p-IKKβ, p-p65 and β-actin. (**D,E**) Columns represented the RQ of p-IKKβ and p-p65 after 4 days of TMP treatment. *P<0.05 vs. control, ^#^*P*<0.01 vs. control. Scale bar, 50 μm.

SA-β-gal staining results indicated the senescence rate of bMSCs in the control group accounting for 23.74%, which was significantly suppressed by TMP treatment (*P<0.05 and ^#^*P*<0.01 vs. control). As exhibited in Figure 4A,B, there was a decrease in SA-β-gal positive cells in the 20, 30, 40, and 50 mg/l TMP treated groups, and particularly in 40 and 50 mg/l TMP groups (^#^*P<*0.01 vs. control), while 60 mg/l TMP exerted little effect on the anti-senescence.

Western blot analysis indicated that 20–50 mg/l TMP could decrease the relative quantity (RQ) of p-IKKβ (^#^*P*<0.01 vs. control; Figure 4C,D). Similar to the change tendency of p-IKKβ RQ, a change pattern in the relative levels of p-p65 was observed along with changes of TMP concentration. In contrast with the control, the RQ of p-p65 was significantly decreased (^#^*P*<0.01 vs. control) when the bMSCs were exposed to 40 and 50 mg/l TMP (Figure 4C,E).

### Measurement of TNF-α and IL-1β contents

ELISA results indicated that the contents of TNF-α and IL-1β in the control group were higher compared with the other groups, which further confirmed the hypothesis that cellular senescence is accompanied with an inflammatory reaction [38]. The inflammatory reaction was alleviated by TMP treatment using the appropriate concentrations. As indicated in Table 2, 30–50 mg/l TMP significantly reduced the amount of these two inflammatory factors (*P<0.05 and ^#^*P*<0.01 vs. control).

**Table 2:** Measurement of TNF-α and IL-1β contents by ELISA (means ± SD, *n*=4)

TMP (mg/l)	TNF-α (ng/l)	IL-1β (ng/l)
0	37.97 ± 1.86	12.47 ± 0.78
20	35.06 ± 1.18	11.04 ± 0.71
30	32.74 ± 0.84*	10.25 ± 0.41*
40	25.29 ± 1.39^†^	8.32 ± 0.49^†^
50	29.65 ± 0.78^†^	9.98 ± 0.25*
60	34.75 ± 1.54	11.07 ± 1.05

* *P* <0.05. ^†^*P*<0.01 vs. control

### Analysis of bMSC proliferation ability

**Figure 5:**
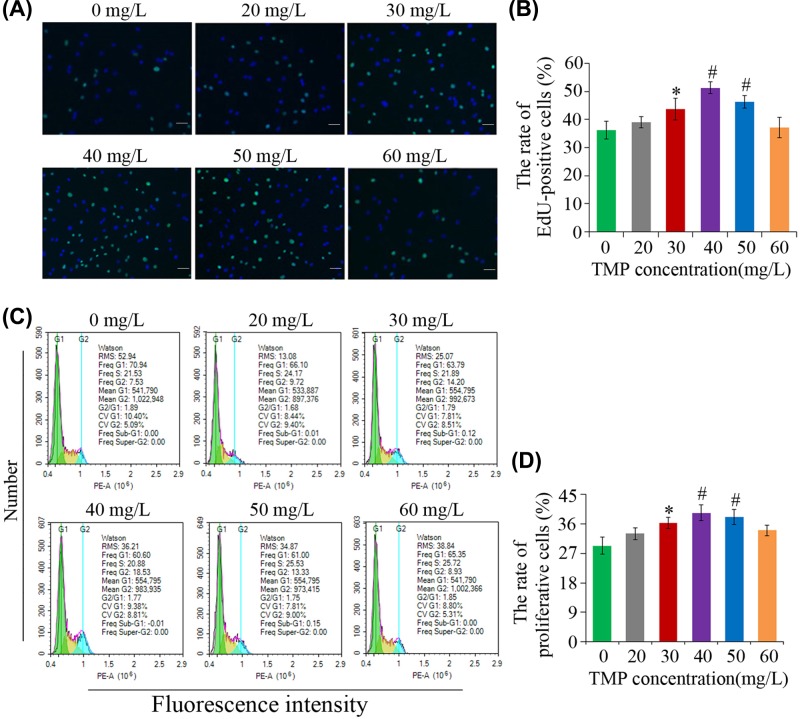
Analysis of TMP effects on proliferation ability of bMSCs and the cell cycles (**A**) Representative fluorescence microscopy images showing EdU staining of MSCs: cells labeled with EdU were green, and the blue ones were labeled by Hoechst33342. (**B**) The statistics of the percentage of EdU+ bMSCs in each group. (**C**) Analysis of cell cycle through FCM. (**D**) The statistics of the percentage of the proliferative cells. *P<0.05 vs. control, ^#^*P*<0.01 vs. control. Scale bar, 50 μm.

Subsequent to incubation with EdU reagent for 24 h, images were captured under a fluorescent microscope (Figure 5A). Green cells were labeled by EdU, and blue cells were labeled by Hoechst33342. As indicated in Figure 5B, the rate of EdU-positive cells was associated with TMP dosage: 40 and 50 mg/l TMP significantly promoted proliferation (^#^*P*<0.01 vs. control); the effect of 30 mg/l TMP was slightly less, however, its EdU-positive rate remained higher compared with the control group (*P<0.05 vs. control); and 20 and 60 mg/l TMP exhibited minimal effect.

FCM analysis indicated that there was an increase in the bMSC percentage in the proliferative (S+G_2_+M) phase following TMP treatment (*P<0.05 and ^#^*P*<0.01 vs. control). In the control group, the proliferation percentage of the cells was only 29.29 ± 2.57%, while TMP treatment for 4 days increased the proliferation rate of bMSCs. Following treatment with 40 mg/l TMP for 4 days, the proliferation percentage increased to 39.33 ± 2.39% (Figure 5C,D), a higher fold-change of ∼1.3, compared with the control group (^#^*P*<0.01 vs. control). No significant differences were observed in the comparison between the 20 or the 60 mg/l TMP group and the control.

### Neuronal differentiation and immunocytochemistry staining

**Figure 6:**
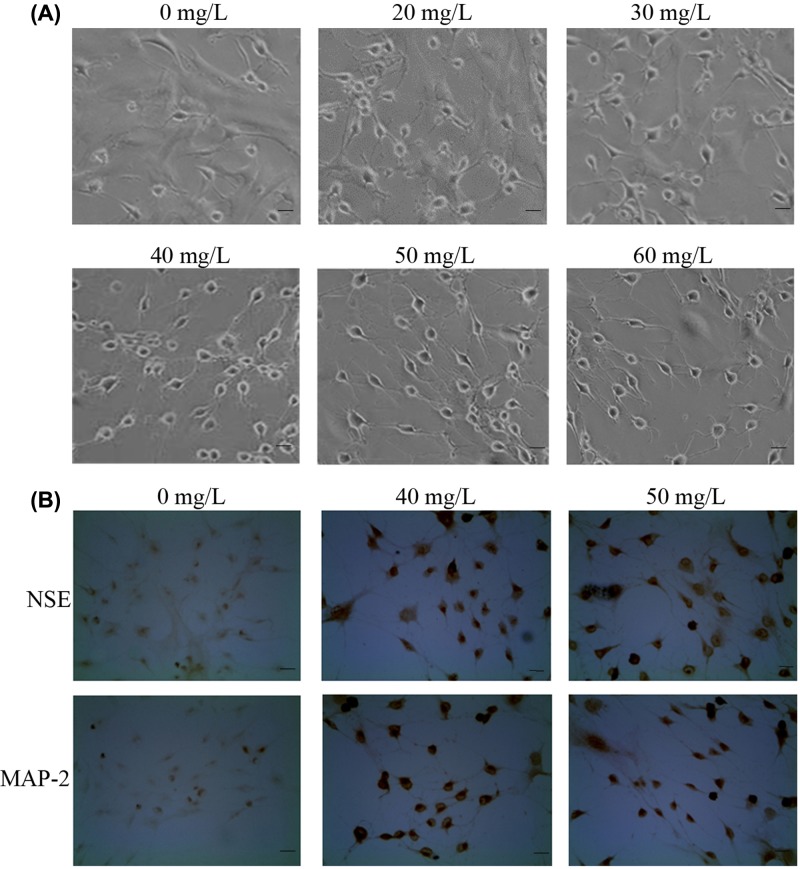
Neural differentiation of bMSCs and immunocytochemistry identification (**A**) Microscopy images showing the morphology of differentiated cells after 7 days of induction. (**B**) Immunocytochemistry staining with antibodies of NSE/MAP-2 was performed to the groups of 40 and 50 mg/l TMP at the 7^th^ day of induction. Scale bar, 50 μm.

Observations of changes in cell morphology were initiated from the first day of neuronal differentiation induction. The pretreatment with 40 and 50 mg/l TMP gave rise to the maximum number of neuron-like cells following 5 days of induction. On the 7^th^ day of induction, the neuronal phenotypes of the differentiated cells in these two groups remained in a good state (Figure 6A). All differentiated cells demonstrated overt neuronal appearance with retracted cell body, refractive karyon and one or more long cytoplasmic processes connected to one another to form network-like structures. On day 3 of induction, the majority of cells in the 60 mg/l TMP group exhibited the aforementioned morphology. However, the protruded dendrites and axons from differentiated cells became shorter between the first 5 and 7 days of induction (Figure 6A). Apoptosis was indicated to take place at later differentiation stages in this group.

After 7 days of neuronal induction, immunocytochemistry staining was performed to identify the differentiated cells in the groups of 0, 40, and 50 mg/l TMP. Compared with the control, almost all differentiated cells in these two groups were positive in both NSE and MAP-2, and appeared hyperchromatic (Figure 6B), which indicated that 4 days of TMP pretreatment at 40 and 50 mg/l, plus 7 days of induction with 20 μg/l NGF + 20 μg/l BDNF, may lead bMSCs to develop into mature neuron-like cells.

### Expression analysis of neuronal bHLH TFs using qRT-PCR

**Figure 7:**
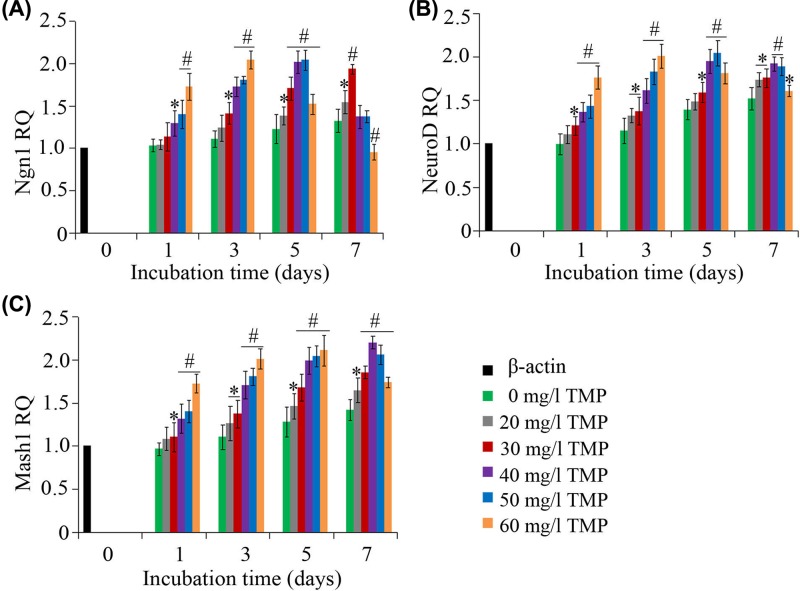
qRT-PCR detection of the change patterns in the expression of bHLH TFs (**A**) The RQ of Ngn1 in different groups at days 1, 3, 5, and 7 after neural induction. (**B**) The RQ of NeuroD in different groups at days 1, 3, 5, and 7 after neural induction. (**C**) The RQ of Mash1 in different groups at days 1, 3, 5, and 7 after neural induction. β-actin served as an endogenous control gene. *P<0.05 vs. control; ^#^*P*<0.05 vs. control.

The qRT-PCR assay was applied to examine the expression changes of Ngn1, NeuroD, and Mash1 during the period of bMSC neuronal differentiation. As indicated in Figure 7A,B, the change of expression patterns in Ngn1 and NeuroD were similar. In the 20–50 mg/l TMP groups, the expression levels of these two TFs gradually increased during the first 5 days of induction, and subsequently dropped on the 7^th^ day of induction. In addition, their expression levels in the 60 mg/l TMP group rapidly reached a peak on day 3 of induction, and declined thereafter. Compared with the control, the expression levels of Mash1 (Figure 7C) were significantly enhanced in all groups during all time points; except the 60 mg/l TMP group, where the Mash1 expression level on the 7^th^ day of induction began to drop, which was believed to be associated with the cell death involved in the later stages of differentiation.

### Western blot verification

**Figure 8:**
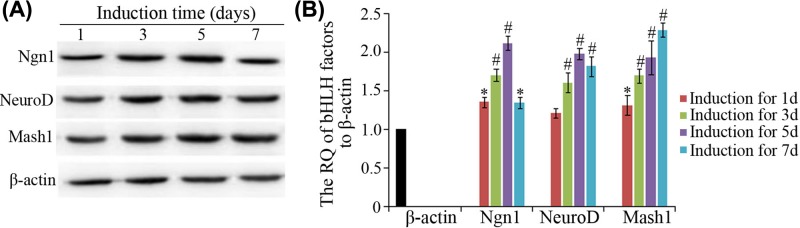
The effects of pre-treatment with 40 mg/l TMP on the expression profiles of three bHLH factors (Ngn1, NeuroD, and Mash1) during neural differentiation (**A**) Western blot results of three bHLH factors on the 1^st^, 3^rd^, 5^th^ and 7^th^ days of induction. (**B**) Columns represented the RQ of Ngn1, NeuroD and Mash1 on the 1^st^, 3^rd^, 5^th^ and 7^th^ days of induction in 40 mg/l TMP group.

To confirm the effects of TMP on the expression changes of three neuronal bHLH factors (Ngn1, NeuroD, and Mash1), the cells, pre-treated with 40 mg/l TMP and induced with NIM for 1, 3, 5, and 7 days, were selected for Western blot verification. The results of Western blot exhibited TMP treatment did affect the expression profiles of these three neuronal bHLH TFs (Figure 8A,B), whose change patterns were similar to that of the qRT-PCR in 40 mg/l TMP group. The relative amount of Ngn1 and NeuroD was in the increasing state in the first 5 days of neural induction, and their quantities were approximately twice as much as that of the control. Mash1 was increasing in its protein content during the induction duration compared with the control, and its amount was increased more than two-times compared with control.

## Discussion

MSCs were verified to possess a wide range of multiple therapeutic properties like tissue regeneration, anti-inflammatory, anti-apoptotic, anti-fibrotic, and immunomodulatory [48]. Therefore, transplantation of MSCs has been considered a promising regenerative strategy for some diseases, such as traumatic brain injuries, spinal cord injury, Parkinson’s disease, and Alzheimer’s disease. However, although MSCs are available from a variety of tissues, the quantity that can be obtained is relatively low. In order to meet the basic requirement of cell therapy protocols, MSCs need expansion *ex vivo* to obtain adequate quantities through successive subculture, which in turn is liable to trigger cell aging. Along with cellular aging, telomere shortening [49,50], proliferation impairment [18], and low differentiation potential [14] have been reported in MSCs. A previous research reported that cellular senescence abrogated the therapeutic potential of MSCs, by inhibiting the lymphocyte-inhibitory activity of MSCs and reducing their migratory capacity in response to pro-inflammatory signals [51]. In addition, once they were transplanted into the lesion sites, a rapid time-dependent decrease in survival rate of the old MSCs engrafted was indicated [52].

Therefore, further investigations in the techniques involved for regulating the self-renewal and lineage commitment of MSCs is highly essential in regenerative medicine. To alleviate the cytotoxicity of chemical agents and reduce the experimental cost [53,54], a number of researchers are focusing on the development of natural drugs, including a variety of herbs and their active ingredients, which have been reported to exhibit numerous protective effects on MSCs in numerous aspects [55]. In light of the beneficial effects of TMP on cells and organs, the present study attempted to use it to enhance the self-renewal and maintain the neuronal differentiation of bMSCs.

On the premise of ensuring cellular viability, the dosage effect of TMP on bMSC senescence was investigated with SA-β-gal staining. TMP dosage between 20 and 50 mg/l decreased the senescence rate of bMSCs. In order to examine the underlying mechanism of the aforementioned observations, the role of TMP was investigated in regulating the activity of NF-κB signaling in P_7_ bMSCs. It has been reported that NF-κB signaling is activated by a biochemical cascade, including cytokines, growth factors, and stress stimuli, and further activates a range of genes involved in immune and inflammatory responses [56], and in a number of physiological processes, including cellular growth, differentiation, and survival [57]. In accordance with previous studies [37–39,58], ELISA results confirmed that senescent bMSCs may secrete abundant pro-inflammatory factors, including TNF-α and IL-1β, and these cytokines in turn activate their downstream NF-κB signaling pathways by binding to their respective receptors, TNFR and IL-1R. The activation of NF-κB signaling contributed to bMSC senescence as indicated in the present study. The secretion capability of TNF-α and IL-1β in P_7_ bMSCs may be suppressed by TMP. The decline of pro-inflammatory factor quantities caused by TMP contributed to inactivation of NF-κB signaling, by which TMP may delay the bMSC senescence phenotype. In fact, the anti-aging effect of TMP had been confirmed *in vivo* experiments [59,60]. For instance, TMP treatment may elevate the activity of choline o-acetyltransferase and acetylcholinesterase (cartwright blood group), and increase muscarinic receptor binding sites in Alzheimer’s disease model mice induced by d-galaclose, as the model mice’s cognitive function, learning and memory were improved [60].

Proliferation assay indicated that 4 days of incubation with 30–50 mg/l TMP significantly enhanced the proliferation capability of bMSCs by promoting them to enter the proliferative stage, while TMP at a concentration of 20 and 60 mg/l exhibited a minimal effect. In accordance with previous research, the effect of TMP on cell proliferation was associated with its dosage, as TMP overdose (>100 ng/ml) inhibited the proliferation of brain endothelial cells [32].

It was revealed that incubation *in vitro* with 30–50 mg/l TMP for 4 days maintained the self-renewal of bMSCs by enhancing cell viability, promoting proliferation, and resisting senescence. The aim of the present study was for the transplanted bMSCs not only to survive and replicate, but also to differentiate into local nerve cells for repairing injury. Therefore, the effects of TMP precondition on the neuronal differentiation of bMSCs were further examined. It was indicated that 40–50 mg/l TMP treatment facilitated neuronal differentiation, verified by presentation of the typical neuronal morphology and expression of the two important neuron-specific markers, MAP-2 and NSE. Meanwhile, the changes in expression of neuronal bHLH factors (Ngn1, NeuroD, and Mash1) were detected in differentiated cells using qRT-PCR and Western blot. As an important type of TFs, the active bHLH proteins can activate their downstream target genes involved in nerve differentiation, by binding to the E box-DNA response element [61], which is crucial in neuronal subtype specification in certain brain regions and in peripheral ganglia. The qRT-PCR results indicated that TMP pretreatment induced these three TFs to produce their individual expression patterns. In 20 and 30 mg/l TMP groups, Ngn1 expression levels were enhanced during the differentiation process, and the expression levels peaked on day 5 following differentiation in the 40 and 50 mg/l TMP groups. The profiles of NeuroD expression were similar to Ngn1s. Consistent with previous studies, proneuronal TFs, including Ngn1 and NeuroD, were mainly expressed in neuronal precursors and immature neurons to promote neurogenesis, and decreased in the late stage of neuronal differentiation of MSCs [62,63]. Mash1 not only promoted neuronal differentiation, but also maintained the neuronal phenotype, and therefore, it was expressed in immature and mature neurons [64,65]. Similar to these studies, the expression levels of Mash1 were increasing throughout the differentiation process in each group, except in the 60 mg/l TMP group where its expression decreased, which may be associated with the anti-aging effect exhibited by 60 mg/l TMP. And Western blot assay further confirmed the results of qRT-PCR. Namely, in 40 mg/l TMP group the change patterns in expression of these three bHLH factors were equal between qRT-PCR and Western blot.

## Conclusion

The concentration TMP at 40 and 50 mg/l significantly enhanced bMSC viability, and delayed the senescence of bMSCs by suppressing the activity of NF-κB signaling and reducing the levels of pro-inflammatory factors, including TNF-α and IL-1β. The anti-aging effect of TMP benefited to enhance the proliferative ability of bMSCs at P_7_ and to maintain their neuronal differentiation potential. The present study highlighted the promising application of TMP in MSCs-based therapy in regenerative medicine for neurological disorders.

## Abbreviations

APC: allophycocyanin
BDNF: brain-derived neurotrophic factor
bHLH: basic helix–loop–helix
bMSC: bone marrow mesenchymal stem cell
CCK-8: cell counting kit-8
EdU: 5-ethynyl-2′-deoxyuridine
ELISA: enzyme-linked immunosorbent assay
FCM: flow cytometry
FITC: fluorescein isothiocyanate
HRP: horseradish peroxidase
L-DMEM: low Dulbecco’s modified Eagle’s medium
IκB: inhibitor of NF-κB
IKK: IκB kinase
IL: interleukin
IL-1R: receptor of interleukin-1
MAP-2: microtubule-associated protein 2
Mash1: mammalian achaete–scute homolog 1
MHC: major histocompatibility complex
MSC: mesenchymal stem cell
NEMO: NF-κB essential modulator
NeuroD: neuronal differentiation 1
NF-κB: nuclear Factor κ-gene Binding
Ngn1: neurogenin 1
NIM: neuronal induction medium
NSE: neuron-specific enolase
PI: propidium iodide
PVDF: polyvinylidene difluoride
P_4_: passage 4
P_7_: passage 7
qRT-PCR: quantitative real-time polymerase chain reaction
RIPA: radio-immunopreciapitation assay
SA-β-gal: senescence-associated β-galactosidase
SD: sprague–dawley
TF: transcription factor
TMP: tetramethylpyrazine
TNF: tumor necrosis factor
TNFR: receptor of tumor necrosis factor
TRADD: TNF receptor associated death domain
TRAF: TNF receptor associated factor

## References

[1] Song X.Q., Su L.N., Wei H.P., Liu Y.H., Yin H.F., Li J.H. (2017) The effect of Id1 gene silencing on the neural differentiation of MSCs. Biotechnol. Biotechnol. Equip. 31, 1–9

[2] Zhang Y., Böse T., Unger R.E., Jansen J.A., Kirkpatrick C.J. and van den Beucken J.J.J.P. (2017) Macrophage type modulates osteogenic differentiation of adipose tissue MSCs. Cell Tissue Res. 69, 273–286 10.1007/s00441-017-2598-8 PMC5552848 28361303

[3] Kim E.Y., Lee K.B., Yu J., Lee J.H., Kim K.J., Han K.W. (2014) Neuronal cell differentiation of mesenchymal stem cells originating from canine amniotic fluid. Hum. Cell 27, 51–58 10.1007/s13577-013-0080-9 24166061 PMC3964299

[4] Jin W., Xu Y.P., Yang A.H. and Xing Y.Q. (2015) In vitro induction and differentiation of umbilical cord mesenchymal stem cells into neuron-like cells by all-trans retinoic acid. Int. J. Ophthalmol. 8, 250–256 25938036 10.3980/j.issn.2222-3959.2015.02.07 PMC4413565

[5] Wu C.G., Zhang J.C., Xie C.Q., Parolini O. and Silini A Huang Y.Z. (2015) In vivo tracking of human placenta derived mesenchymal stem cells in nude mice via 14C-TdR labeling. BMC Biotech. 15, 1–9 10.1186/s12896-015-0174-4 26070459 PMC4465458

[6] Allickson J.G., Sanchez A., Yefimenko N., Borlongan C.V., Sanberg P.R. and Yefimenko N. (2011) Recent studies assessing the proliferative capability of a novel adult stem cell identified in menstrual blood. Open Stem Cell J. 3, 4–10 10.2174/1876893801103010004 21686032 PMC3113522

[7] Guo L., Wang L., Wang L., Yun-Peng S., Zhou J.J., Zhao Z. (2017) Resveratrol induces differentiation of human umbilical cord mesenchymal stem cells into neuron-Like cells. Stem Cells Int. 2017, 1–7 10.1155/2017/1651325 PMC5415670 28512471

[8] Park S., Choi Y., Jung N., Kim J., Oh S., Yu Y. (2017) Autophagy induction in the skeletal myogenic differentiation of human tonsil-derived mesenchymal stem cells. Int. J. Mol. Med. 39, 831–840 10.3892/ijmm.2017.2898 28259927 PMC5360438

[9] Zhou X., Cui L.N., Zhou X.M., Yang Q., Wang L., Guo G.Y. (2017) Induction of hepatocyte-like cells from human umbilical cord-derived mesenchymal stem cells by defined microRNAs. J. Cell. Mol. Med. 21, 881–893 27874233 10.1111/jcmm.13027 PMC5387126

[10] Lu Y.S., Zhou Z.H., Tao J., Dou B., Gao M.J. and Liu Y. (2014) Overexpression of stearoyl-CoA desaturase 1 in bone marrow mesenchymal stem cells enhance the expression of induced endothelial cells. Lipids Health Dis. 13, 53–61 10.1186/1476-511X-13-53 24650127 PMC3974181

[11] Chan J., O’Donoghue K., De la Fuente J., Roberts I.A., Kumar S., Morgan J.E. (2005) Human fetal mesenchymal stem cells as vehicles for gene delivery. Stem Cells 23, 93–102 10.1634/stemcells.2004-0138 15625126

[12] Matyas J.J., Stewart A.N., Goldsmith A., Nan Z., Skeel R.L., Rossignol J. (2017) Effects of bone-marrow–derived MSC transplantation on functional recovery in a rat model of spinal cord injury: comparisons of transplant locations and cell concentrations. Cell Transplant. 26, 1472–1482 10.1177/0963689717721214 28901182 PMC5680979

[13] Li J.R. and Qu T.T. (2017) Into the eyes of bone marrow-derived mesenchymal stem cells therapy for myocardial infarction and other diseases. Stem Cell Investig. 4, 69–73 10.21037/sci.2017.08.01 28920062 PMC5590020

[14] Lee J.K., Jin H.K., Endo S., Schuchman E.H., Carter J.E. and Bae J.S. (2010) Intracerebral transplantation of bone marrow-derived mesenchymal stem cells reduces amyloid-Beta deposition and rescues memory deficits in Alzheimer’s disease mice by modulation of immune responses. Stem Cells 28, 329–343 20014009 10.1002/stem.277

[15] Yoon D.S., Kim Y.H., Jung H.S., Paik S. and Lee J.W. (2011) Importance of Sox2 in maintenance of cell proliferation and multipotency of mesenchymal stem cells in low-density culture. Cell Prolif. 44, 428–440 10.1111/j.1365-2184.2011.00770.x 21951286 PMC6495637

[16] Ksiazek K. (2009) A comprehensive review on mesenchymal stem cell growth and senescence. Rejuvenation Res. 12, 105–116 10.1089/rej.2009.0830 19405814

[17] Wang X.X., Ma S.S., Meng N., Yao N., Zhang K., Li Q.H. (2016) Resveratrol exerts dosage-dependent effects on the self-renewal and neural differentiation of hUC-MSCs. Molecules Cells 39, 418–425 27109421 10.14348/molcells.2016.2345 PMC4870190

[18] Gao B., Lin X.S., Jing H., Fan J., Ji C.C. and Jie Q. (2018) Local delivery of tetramethylpyrazine eliminates the senescent phenotype of bone marrow mesenchymal stromal cells and creates an anti-inflammatory and angiogenic environment in aging mice. Aging Cell 17, e12741 10.1111/acel.12741 29488314 PMC5946084

[19] Guo M., Liu Y. and Shi D.Z. (2016) Cardiovascular actions and therapeutic potential of tetramethylpyrazine (active component isolated from rhizoma chuanxiong): roles and mechanisms. Biomed. Res. Int. 2016, 2430329 10.1155/2016/2430329 27314011 PMC4893570

[20] Li Y., Song P., Zhu Q., Yin Q.Y., Ji J.W., Li W. (2014) Liguzinediol improved the heart function and inhibited myocardial cell apoptosis in rats with heart failure. Acta Pharmacol. Sin. 35, 1257–1264 10.1038/aps.2014.75 25220638 PMC4186991

[21] Kuang X., Zhou H.J., Thorne A.H., Chen X.N., Li L.J. and Du J.R. (2017) Neuroprotective effect of Ligustilide through induction of α-Secretase processing of both APP and Klotho in a mouse model of Alzheimer’s ddisease. Front. Aging Neurosci. 9, 353–365 10.3389/fnagi.2017.00353 29163135 PMC5673635

[22] Wu W., Yu X., Luo X.P., Yang S.H. and Zheng D. (2013) Tetramethylpyrazine protects against scopolamine-induced memory impairments in rats by reversing the cAMP/PKA/CREB pathway. Behav. Brain Res. 253, 212–216 10.1016/j.bbr.2013.07.052 23916742

[23] Lu C., Zhang J., Shi X.X., Miao S., Bi L.L. and Zhang S. (2014) Neuroprotective effects of tetramethylpyrazine against dopaminergic neuron injury in a rat model of Parkinson’s disease induced by MPTP. Int. J. Biol. Sci. 10, 350–357 10.7150/ijbs.8366 24719552 PMC3979987

[24] Xiong L., Fang Z.Y., Tao X.N., Bai M. and Feng G. (2007) Effect and mechanism of ligustrazine on th1/th2 cytokines in a rat asthma model. Am. J. Chin Med. 35, 1011–1020 10.1142/S0192415X07005478 18186587

[25] Wang J.Y., Chen W.M., Wen C.S., Huang S.C., Chen P.W. and Chiu J.H. (2016) Du-Huo-Ji-Sheng- Tang and its active component *Ligusticum chuanxiong* promote osteogenic differentiation and decrease the aging process of human mesenchymal stem cells. J. Ethnopharmacol. 198, 64–72 10.1016/j.jep.2016.12.011 28040510

[26] Li L., Chu L.S., Fang Y., Yang Y., Qu T.B., Zhang J.P. (2017) Preconditioning of bone marrow-derived mesenchymal stromal cells by tetramethylpyrazine enhances cell migration and improves functional recovery after focal cerebral ischemia in rats. Stem Cell Res. Ther. 8, 112–121 28499457 10.1186/s13287-017-0565-7 PMC5429508

[27] Zhang L., Deng M.Y. and Zhou S.W. (2011) Tetramethylpyrazine inhibits hypoxia-induced pulmonary vascular leakage in rats via the ROS-HIF-VEGF pathway. Pharmacology 87, 265–273 10.1159/000326082 21494058

[28] Xiao Z.M., Hu J.Z., Lv H.B., Zhuo X.L., Xu D.Q., Wang S.X. (2012) The effect of tetramethylpyrazine on the expression of NF-κB and IκBα after acute spinal cord injury in the rat model. Basic Clin. Med. 32, 407–412

[29] Zhang M.S., Gao F., Teng F.M. and Zhang C.B. (2014) Tetramethylpyrazine promotes the proliferation and migration of brain endothelial cells. Mol. Med. Rep. 10, 29–32 10.3892/mmr.2014.2169 24789060 PMC4068727

[30] Nan C.R., Guo L., Zhao Z.M., Ma S.C., Liu J.X., Yan D.D. (2016) Tetramethylpyrazine induces differentiation of human umbilical cord-derived mesenchymal stem cells into neuron-like cells in vitro. Int. J. Oncol. 48, 2287–2294 10.3892/ijo.2016.3449 27035275 PMC4863923

[31] Gao X., Zhao X.L., Zhu Y.H., Li X.M., Xu Q., Lin H.D. (2011) Tetramethylpyrazine protects palmitate-induced oxidative damage and mitochondrial dysfunction in C2C12 myotubes. Life Sci. 30, 803–809 10.1016/j.lfs.2011.02.025 21396380

[32] Bi L., Yan X.J., Chen W.P., Gao J., Qian L. and Qiu S. (2016) Antihepatocellular carcinoma potential of Tetramethylpyrazine induces cell cycle modulation and mitochondrial-dependent apoptosis: regulation of p53 signaling pathway in HepG2 cells in vitro. Integr. Cancer Ther. 15, 226–236 10.1177/1534735416637424 27179035 PMC5736061

[33] Shen J.L., Zeng L.W., Pan L.M., Yuan S.F., Wu M. and Kong X.D. (2018) Tetramethylpyrazine regulates breast cancer cell viability, migration, invasion and apoptosis by affecting the activity of Akt and caspase-3. Oncol. Lett. 15, 4557–4563 29541225 10.3892/ol.2018.7851 PMC5835919

[34] Moiseeva O., Deschênes-Simard X., St-Germain E., Igelmann S., Huot G., Cadar A.E. (2013) Metformin inhibits the senescence-associated secretory phenotype by interfering with IKK/NF-κB activation. Aging Cell 12, 489–498 10.1111/acel.12075 23521863

[35] Zeng Y., Wang P.H., Zhang M. and Du J.R. (2016) Aging-related renal injury and inflammation are associated with downregulation of Klotho and induction of RIG-I/NF-κB signaling pathway in senescence-accelerated mice. Aging Clin. Exp. Res. 28, 69–76 25986237 10.1007/s40520-015-0371-y

[36] Zaidi A.H. and Manna S.K. (2016) Profilin-PTEN interaction suppresses NF-κB activation via inhibition of IKK phosphorylation. Biochem. J. 473, 859–872 10.1042/BJ20150624 26787927

[37] Li Q., Antwerp D.V., Mercurio F., Lee K.F. and Verma I.M. (1999) Severe liver degeneration in mice lacking the IκB kinase 2 gene. Science 284, 321–325 10.1126/science.284.5412.321 10195897

[38] Li F., Zhang J., Arfuso F., Chinnathambi A., Zayed M.E., Alharbi S.A. (2015) NF-κB in cancer therapy. Arch. Toxicol. 89, 711–721 10.1007/s00204-015-1470-4 25690730

[39] Zhao J. (2016) A Causal Role of ATM- and NEMO-Dependent NF-κB Activation in DNA Damage-Induced Senescence and Aging, University of Pittsburgh, d-scholarship.pitt.edu

[40] Shin E.Y., Hay H.S., Lee M.H., Goh J.N., Tan T.Z., Sen Y.P. (2014) DEAD-box helicase DP103 defines metastatic potential of human breast cancers. J. Clin. Invest. 124, 3807–3824 10.1172/JCI73451 25083991 PMC4151228

[41] Li C.X., Lo C.M., Lian Q.Z., Ng K.T.P., Liu X.B. and Ma Y.Y. (2016) Repressor and activator protein accelerates hepatic ischemia reperfusion injury by promoting neutrophil inflammatory response. Oncotarget 7, 27711–27723 27050284 10.18632/oncotarget.8509 PMC5053682

[42] Zhang Y., Chiu S.M., Liang X., Gao F., Zhang Z., Liao S. (2015) Rap1-mediated nuclear factor-kappaB (NF-κB) activity regulates the paracrine capacity of mesenchymal stem cells in heart repair following infarction. Cell Death Discov. 1, 15007–15017 10.1038/cddiscovery.2015.7 27551443 PMC4981000

[43] Cai Y., Sukhova G.K., Wong H.K., Xu A.M., Tergaonkar V., Vanhoutte P.M. (2015) Rap1 induces cytokine production in pro-inflammatory macrophages through NFκB signaling and is highly expressed in human atherosclerotic lesions. Cell Cycle 14, 3580–3592 10.1080/15384101.2015.1100771 26505215 PMC4825742

[44] Huang L.H., Yang H., Su X., Gao Y.R., Xue H.N. and Wang S.H. (2017) Neodymium oxide induces cytotoxicity and activates NF-κB and caspase-3 in NR8383 cells. Biomed. Environ. Sci. 30, 75–78 28245903 10.3967/bes2017.010

[45] Song X.Q., Su L.N., Yin H.F., Dai J. and Wei H.P. (2018) Effects of HSYA on the proliferation and apoptosis of MSCs exposed to hypoxic and serum deprivation conditions. Exp. Ther. Med. 15, 5251–5260 29904409 10.3892/etm.2018.6125 PMC5996714

[46] Lee B.Y., Han J.A., Im J.S., Morrone A., Johung K., Goodwin E.C. (2006) Senescence- associated β-galactosidase is lysosomal β-galactosidase. Aging Cell 5, 187–195 10.1111/j.1474-9726.2006.00199.x 16626397

[47] Livak K.J. and Schmittgen T.D. (2001) Analysis of relative gene expression data using real-time quantitative PCR and the 2(-Delta Delta C (T)) method. Methods 25, 402–408 10.1006/meth.2001.1262 11846609

[48] Caplan A. (2009) Why are MSCs therapeutic? New data: new insight. J. Pathol. 217, 318–324 10.1002/path.2469 19023885 PMC8793150

[49] Castorina A., Szychlinska M.A., Marzagalli R. and Musumeci G. (2015) Mesenchymal stem cells-based therapy as a potential treatment in neurodegenerative disorders: is the escape from senescence an answer? Neural Regen. Res. 10, 850–858 10.4103/1673-5374.158352 26199588 PMC4498333

[50] Khattar E., Kumar P., Liu C.Y., Akıncılar S.C., Raju A., Lakshmanan M. (2016) Telomerase reverse transcriptase promotes cancer cell proliferation by augmenting tRNA expression. J. Clin. Invest. 126, 4045–4060 10.1172/JCI86042 27643433 PMC5096818

[51] Sepúlveda J.C., Tomé M., Fernández M.E., Delgado M., Campisi J., Bernad A. (2014) Cell senescence abrogates the therapeutic potential of human mesenchymal stem cells in the lethal endotoxemia model. Stem Cells 32, 1865–1877 10.1002/stem.1654 24496748 PMC4209016

[52] Li L., Guo Y.F., Zhai H.X., Yin Y.X., Zhang J.J., Chen H.W. (2014) Aging increases the susceptivity of MSCs to reactive oxygen species and impairs their therapeutic potency for myocardial infarction. PLoS ONE 9, e111850 10.1371/journal.pone.0111850 25393016 PMC4230939

[53] Li D., Wang G.Y., Dong B.H., Zhang Y.C., Wang Y.X. and Sun B.C. (2007) Biological characteristics of human placental mesenchymal stem cells and their proliferative response to various cytokines. Cells Tissues Organs 186, 169–179 10.1159/000105674 17630477

[54] Ikhapoh I.A., Pelham C.J. and Agrawal D.K. (2015) Atherogenic cytokines regulate VEGF-A-induced differentiation of bone marrow-derived mesenchymal stem cells into endothelial cells. Stem Cells Int. 2015, 498328 10.1155/2015/498328 26106428 PMC4464597

[55] Ali F.F. and Hasan T. (2012) Phlorotannin-incorporated mesenchymal stem cells and their promising role in osteogenesis imperfect. J. Med. Hypotheses Ideas 6, 85–89 10.1016/j.jmhi.2012.09.002

[56] Wan F. and Lenardo M.J. (2009) Specification of DNA binding activity of NF-kappaB proteins. Cold Spring Harb. Perspect. Biol. 1, a000067 10.1101/cshperspect.a000067 20066093 PMC2773628

[57] Hilgendorff A., Muth H., Parviz B., Staubitz A., Haberbosch W., Tillmanns H. (2013) Statins differ in their ability to block NF-kappaB activation in human blood monocytes. Int. J. Clin. Pharmacol. Ther. 41, 397–401 10.5414/CPP41397 14518599

[58] Sethi G., Shanmugam M.K., Ramachandran L., Kumar A.P. and Tergaonkar V. (2012) Multifaceted link between cancer and inflammation. Biosci. Rep. 32, 1–15 10.1042/BSR20100136 21981137

[59] Raina A.K., Pardo P., Rottkamp C.A., Zhu X.W., Pereira-Smith O.M. and Smith M.A. (2001) Neurons in Alzheimer disease emerge from senescence. Mech. Ageing Dev. 123, 3–9 10.1016/S0047-6374(01)00333-5 11640946

[60] Zhang C., Wang S.Z. and Wang T. (2008) Effects of Tetramethylpyrazine on the hippocampal cholinergic system in D-galactose induced mice model with Alzheimer’s disease. J. Capital Med. Univ. 29, 15–18

[61] Zhu G.X., Wu Y.X. and Qiao L. (2012) Expression of neurogenin2 and math6 in the embryonal and postnatal mouse inner ear and their molecular regulation. Chin. J. Otology 10, 96–100

[62] Cardozo A.J., Gomez D.E. and Argibay P.F. (2012) Neurogenic differentiation of human adipose-derived stem cells: relevance of different signaling molecules, transcription factors, and key marker genes. Gene 511, 427–436 10.1016/j.gene.2012.09.038 23000064

[63] Velkey J.M. and O’Shea K.S. (2013) Expression of Neurogenin 1 in mouse embryonic stem cells directs the differentiation of neuronal precursors and identifies unique patterns of down-stream gene expression. Dev. Dyn. 242, 230–253 10.1002/dvdy.23920 23288605 PMC4646168

[64] Liu F.F., Xuan A.G., Chen Y., Zhang J.D., Xu L.P., Yan Q.J. (2014) Combined effect of nerve growth factor and brain-derived neurotrophic factor on neuronal differentiation of neural stem cells and the potential molecular mechanisms. Mol. Med. Rep. 10, 1739–1745 10.3892/mmr.2014.2393 25051506 PMC4148384

[65] Ding D.F., Xu L.Q., Xu H., Li X.F., Liang Q.Q., Zhao Y.J. (2014) Mash1 efficiently reprograms rat astrocytes into neurons. Neural Regen. Res. 9, 25–32 10.4103/1673-5374.125326 25206740 PMC4146312

